# Surgical-Orthodontic Treatment of a Severe Hyperdivergent Skeletal Class III Malocclusion With Dento-Alveolar Compensations: A Case Report

**DOI:** 10.7759/cureus.111800

**Published:** 2026-06-30

**Authors:** Mohamed Drissi Lahsini, Nabil Loudini, Fatima Zaoui, Abdelali Halimi

**Affiliations:** 1 Orthodontics and Dentofacial Orthopedics, Faculty of Dental Medicine, Mohammed V University, Rabat, MAR

**Keywords:** dento-alveolar compensation, hyperdivergence, maxillary endognathia, “orthognathic surgery”, skeletal class iii malocclusion

## Abstract

Skeletal Class III malocclusion resulting from maxillary retrognathism is associated with maxillary endognathia, dento-alveolar compensations, and an anterior crossbite and may be complicated by hyperdivergent facial growth patterns. We report the case of a 16-year-old male patient who presented with skeletal Class III malocclusion secondary to maxillary retrognathism and maxillary endognathia, associated with maxillary proalveolia, mandibular retroalveolia, an anterior open bite tendency, vertical hyperdivergence, and a Class III molar and canine relationship bilaterally. The previous extraction of mandibular second premolars significantly complicated space management and pre-surgical decompensation.

Cephalometric analysis showed a skeletal discrepancy (ANB = -3°, AoBo = -8 mm, FMA = 47°), and a difficulty index calculation placed the case in the severe category. Treatment combined pre-surgical orthodontic decompensation, extraction of 14, 25, 18, and 38, maxillary expansion, and combined orthognathic surgery consisting of a Le Fort I osteotomy with advancement and posterior impaction of the maxilla, together with a bilateral sagittal split osteotomy (BSSO) (Obwegeser-Dalpont technique) for mandibular setback, followed by post-surgical orthodontic finishing, allowing the achievement of a satisfactory Class I occlusion at the completion of treatment.

This case demonstrates the importance of comprehensive cephalometric and dento-alveolar assessment in planning combined surgical-orthodontic treatment.

## Introduction

Skeletal Class III malocclusion is a complex craniofacial deformity that may result from maxillary retrognathism, mandibular prognathism, or a combination of both, and is frequently accompanied by transverse maxillary deficiency (endognathia) and dento-alveolar compensations such as maxillary incisor proclination (proalveolia) and mandibular incisor retroclination [1,2]. When the skeletal discrepancy is severe and associated with vertical hyperdivergence, camouflage orthodontic treatment is generally insufficient, and a combined surgical-orthodontic approach becomes the treatment of choice [3].

The architectural severity of a Class III malocclusion can be quantified using comprehensive sagittal, vertical, and dento-alveolar cephalometric parameters, helping to determine whether orthodontic camouflage or orthognathic surgery is indicated [4]. In hyperdivergent Class III patients, the combination of maxillary advancement with posterior impaction (autorotation of the mandible) and mandibular setback via bilateral sagittal split osteotomy (BSSO) allows simultaneous correction of the sagittal discrepancy and control of the vertical dimension [5,6].

A further complicating factor in the present case was the prior extraction of both mandibular second premolars (35 and 45) before the patient's referral, which had to be integrated into the space analysis and overall treatment plan. We report the diagnostic work-up, treatment planning, and surgical-orthodontic management of a 16-year-old male patient who presented with severe hyperdivergent skeletal Class III malocclusion associated with maxillary retrognathism, endognathia, and dento-alveolar compensations. The report follows the CARE guidelines for case reports.

## Case presentation

A 16-year-old male patient presented to the Department of Orthodontics and Dentofacial Orthopedics with a chief complaint of an anterior crossbite and an unsatisfactory facial profile ("Class III" appearance). The medical history was unremarkable. Functional examination revealed mixed (oronasal) breathing, which was clinically diagnosed through a habitual open-mouth posture and further corroborated by a narrowed nasopharyngeal airway space on the lateral cephalometric radiograph.

The patient presented with a long, oval, and symmetric face and an increased lower facial height (Figure 1a). The nasolabial folds were flattened. The smile was dysgracious, exposing both the maxillary and mandibular dentition, and the smile line did not pass at the level of the gingival margins of the maxillary teeth (Figure 1b). In profile, the patient showed a concave facial profile, a reduced cervico-mental distance, an effaced labio-mental sulcus, and a normal nasolabial angle (Figure 1c).

**Figure 1:**
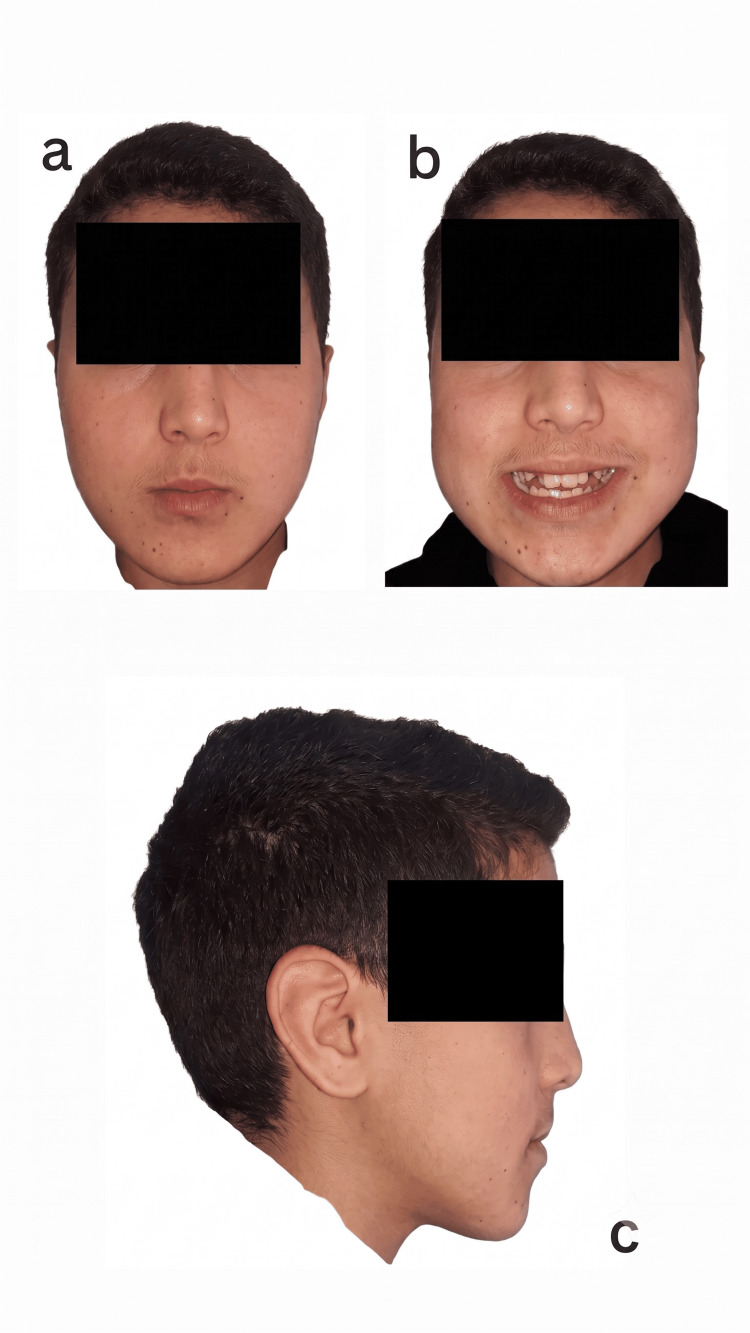
Pre-treatment extraoral photographs. (a) Frontal view at rest, (b) smile, (c) right profile. The patient provided written and signed consent, allowing publication of this identifiable facial image in an open-access journal.

Dental notation follows the World Dental Federation (FDI) two-digit system.

Inter-arch examination revealed a bilateral full-step Class III molar relationship (Figures 2a, 2b), an anterior crossbite with negative overjet, a transverse discrepancy with the maxilla failing to encompass the mandible, a 1 mm deviation of the upper and lower interincisal midlines to the right relative to the facial midline, and a negative vertical overlap indicating an anterior open bite tendency (Figure 2c).

**Figure 2:**
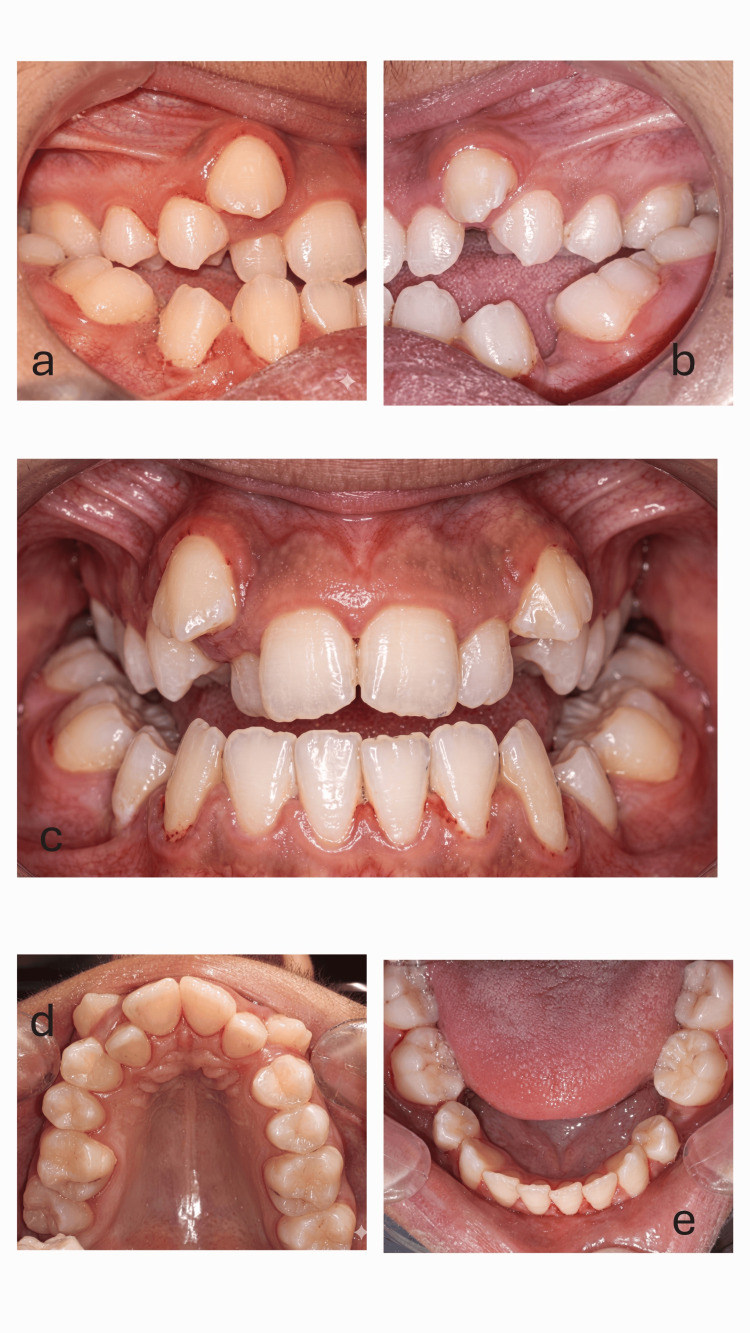
Pre-treatment (T0) intraoral views. (a, b) Right and left lateral occlusal views showing a bilateral Class III molar relationship, (c) frontal occlusal view, (d) maxillary occlusal view (horseshoe-shaped arch, ectopic 13/23), and (e) mandibular occlusal view (U-shaped arch, absence of 35 and 45, mild crowding).

Intra-arcade examination of the maxilla showed a horseshoe-shaped (V-shaped) arch, a deep palatal vault, maxillary endognathia (transverse maxillary deficiency), ectopic positions of teeth 13 and 23, palatal positioning of teeth 12 and 22, and rotations of teeth 16 and 26 (Figure 2d). The mandibular arch was U-shaped, with the absence of teeth 35 and 45, which had been previously extracted, and mild incisor crowding (Figure 2e).

The panoramic radiograph showed a complete permanent dentition, the absence of teeth 35 and 45 due to previous extractions, and the presence of two third molars (18 and 38), with no evidence of periapical or periodontal pathology (Figure 3a). The lateral cephalogram showed a concave facial profile with an increased lower anterior facial height and a steep mandibular plane, consistent with severe vertical hyperdivergence (Figure 3b).

**Figure 3:**
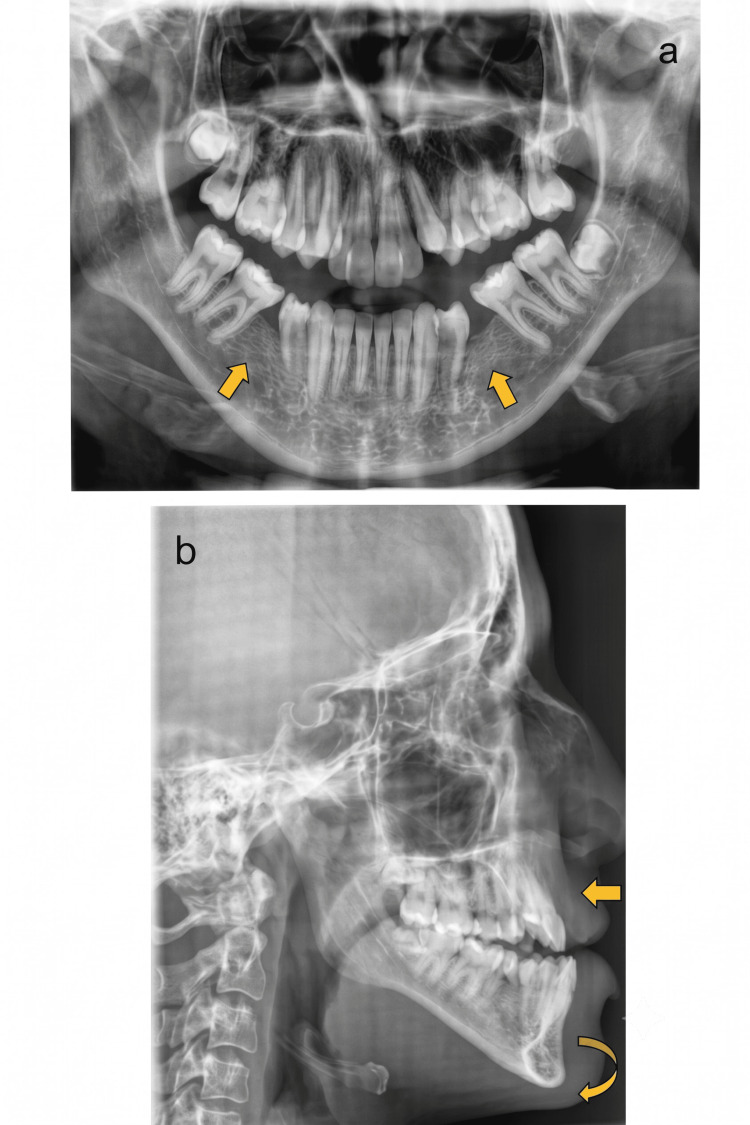
Pre-treatment (T0) radiographic examination. (a) Panoramic radiograph. The arrows on the panoramic radiograph denote the sites of the pre-existing extractions of the mandibular second premolars (teeth 35 and 45), highlighting the structural spaces integrated into the decompensation plan. (b) Lateral cephalogram. The arrows on the lateral cephalogram point to the severe skeletal discrepancy landmarks, specifically emphasizing the steep mandibular plane angle, the increased lower anterior facial height, and the severe hyperdivergent skeletal pattern.

Cephalometric analysis confirmed a skeletal Class III pattern (ANB = -3°, AoBo = -8 mm) due to maxillary retrognathism (SNA = 79°) with a normognathic mandible (SNB = 82°), associated with severe vertical hyperdivergence (FMA = 47°, GoGn-SN = 41°) (Table 1). Dento-alveolar compensations were characterized by maxillary incisor proclination (proalveolia, 1 to NA = 8 mm / 27°) and significant mandibular incisor retroclination (linguoversion, IMPA = 75°), reflecting the natural compensation within the symphysis.

**Table 1:** Cephalometric analysis at T0 (pre-treatment).

Cephalometric variable	Norm (mean ± SD)	T0 (pre-treatment)
SNA (°)	82 ± 2	79°
SNB (°)	80 ± 2	82°
ANB (°)	2 ± 2	-3°
AoBo (mm)	0 ± 2	-8 mm
1 to NA (°)	22°	27°
1 to NA (mm)	4 mm	8 mm
1 to NB (°)	25°	22°
1 to NB (mm)	4 mm	6 mm
1 to 1 (°)	131°	134°
Pog to NB (mm)	0 mm	0 mm
GoGn-SN (°)	32 ± 5	41°
FMA (°)	25 ± 3	47°
FMIA (°)	67 ± 3	58°
IMPA (°)	88 ± 3	75°
Angle Z (°)	73°	66°

A comprehensive mandibular space analysis was performed to evaluate the total arch-length discrepancy, taking into account the preexisting extraction spaces of teeth 35 and 45. The evaluation incorporated anterior crowding, necessary curve of Spee leveling, and the substantial space required for dental decompensation and sagittal Class III correction. This overall assessment quantified a significant mandibular arch-length deficiency, highlighting the clinical necessity of fully utilizing the residual extraction spaces to achieve optimal incisor positioning and correct the intermaxillary discrepancy.

Consequently, the final diagnosis was formulated as a skeletal Class III malocclusion due to maxillary retrognathism and maxillary endognathia, with a normognathic mandibular position and severe vertical hyperdivergence. This skeletal discrepancy was accompanied by dento-alveolar compensation in the form of maxillary proalveolia and mandibular retroalveolia, bilateral Class III canine and molar relationships, an anterior crossbite, and a 23 mm mandibular arch-length discrepancy associated with the previous extraction of teeth 35 and 45.

Given the severe sagittal skeletal discrepancy, the hyperdivergent vertical growth pattern, and the complexity of the dento-alveolar compensations, the patient's condition was classified as highly severe. These accumulated structural imbalances strongly supported the definitive indication for a combined surgical-orthodontic treatment plan over orthodontic camouflage.

The primary treatment objectives were directed at correcting the sagittal skeletal Class III discrepancy through combined maxillary advancement and mandibular setback, while simultaneously controlling and reducing the vertical hyperdivergence through posterior maxillary impaction. Transversally, the goal was to correct the maxillary endognathia through pre-surgical maxillary expansion (disjunction). Additionally, treatment aimed to decompensate the maxillary and mandibular incisor inclinations to allow optimal surgical movements, manage the existing arch-length discrepancy (including the previously extracted 35 and 45 sites), achieve a Class I canine and molar relationship with normal overjet and overbite, and ultimately improve facial esthetics and the labio-mental relationship.

Treatment was carried out in three distinct phases: a pre-surgical orthodontic phase, a surgical phase, and a post-surgical orthodontic finishing phase, as summarized in Table 2.

**Table 2:** Summary of the three treatment phases.

Timepoint	Clinical event
T0 (Feb 2024)	Initial consultation, full clinical and radiographic records, diagnosis, and treatment planning.
Pre-surgical phase	Oral hygiene reinforcement, scaling and polishing; extraction of 14, 25, 18, and 38; placement of a maxillary expander (disjunction); pre-surgical alignment, leveling, and decompensation; space closure.
Surgery (Oct 2024)	Combined orthognathic surgery: Le Fort I osteotomy with maxillary advancement and posterior impaction, and bilateral sagittal split osteotomy (Obwegeser-Dalpont technique) with mandibular setback.
Post-surgical orthodontics	Intercuspation and finishing mechanics.
T1 (Apr 2026)	Debonding, retention, and final clinical and radiographic evaluation.

The pre-surgical orthodontic phase began with patient motivation for strict oral hygiene, scaling, and polishing. Teeth 14, 25, 18, and 38 were extracted to provide the required dental space and to clear the third molars from the planned surgical fields. To correct the transverse maxillary deficiency (endognathia), a rapid maxillary expansion (RME) protocol was initiated using a tooth-borne Hyrax-type disjunctor (Figure 4a). The activation schedule consisted of two quarter-turns per day (0.5 mm/day) for an active expansion period of approximately 14 days. The expansion screw was then locked, and the appliance was maintained in situ for a six-month passive retention phase to allow for adequate bone consolidation of the midpalatal suture. Pre-surgical alignment and leveling were then carried out with full decompensation of the maxillary and mandibular incisor inclinations. Maxillary alignment and leveling were initiated with a 0.012-inch nickel-titanium (NiTi) archwire, allowing the opening of an interincisal diastema; the maxillary canines (13 and 23) and the lateral incisor (12) were intentionally left unengaged in the archwire at this initial stage (Figure 4b).

**Figure 4:**
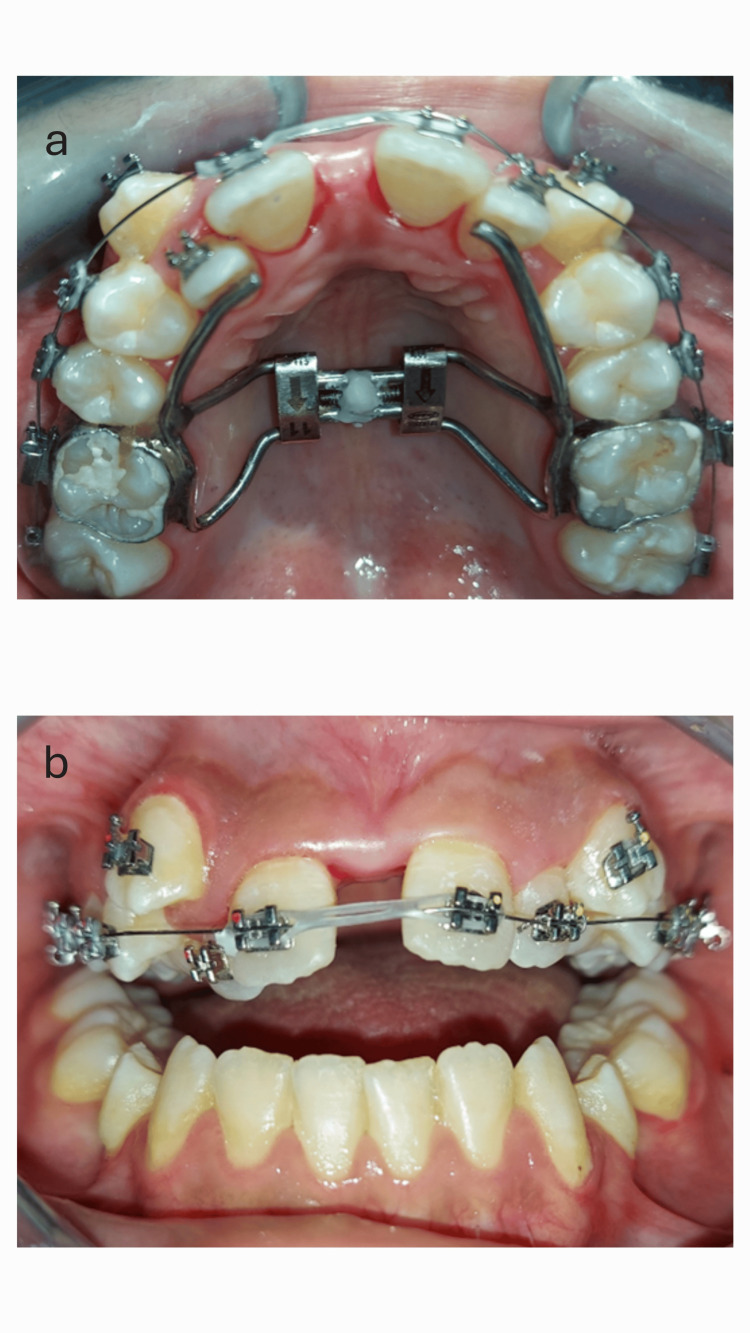
Pre-surgical orthodontic phase. (a) Maxillary expander (disjunctor) in place for correction of maxillary endognathia, and (b) maxillary occlusal view showing opening of the interincisal diastema and alignment/leveling with a 0.012-inch NiTi archwire, with teeth 13, 23, and 12 not yet engaged.

Following the extraction of teeth 14 and 25, space closure was managed using closing ligatures on the maxillary canines (13 and 23), while space was simultaneously opened to bond tooth 12 and bring it progressively into the arch (Figure 5a). Canine retraction mechanics and space management at the 14 and 25 extraction sites were closely monitored (Figures 5b, 5c). Archwire progression followed a rigorous sequential protocol: 0.012-inch, 0.014-inch, 0.016-inch, and 0.018-inch round NiTi archwires, followed by 0.016 × 0.022-inch NiTi and 0.016 × 0.022-inch stainless steel rectangular archwires. Simultaneously, pre-surgical orthodontic preparation of the mandibular arch was performed using a similar sequential archwire progression (from 0.012-inch NiTi to 0.018 × 0.025-inch stainless steel). The mechanics focused on leveling the mandibular teeth and completely utilizing the pre-existing extraction spaces of teeth 35 and 45 to achieve optimal incisor decompensation (retroclination) and align the lower arch, ensuring a coordinated and passive fit with the maxillary arch prior to orthognathic surgery. Pre-surgical preparation was completed on a stable 0.018 × 0.025-inch stainless steel archwire.

**Figure 5:**
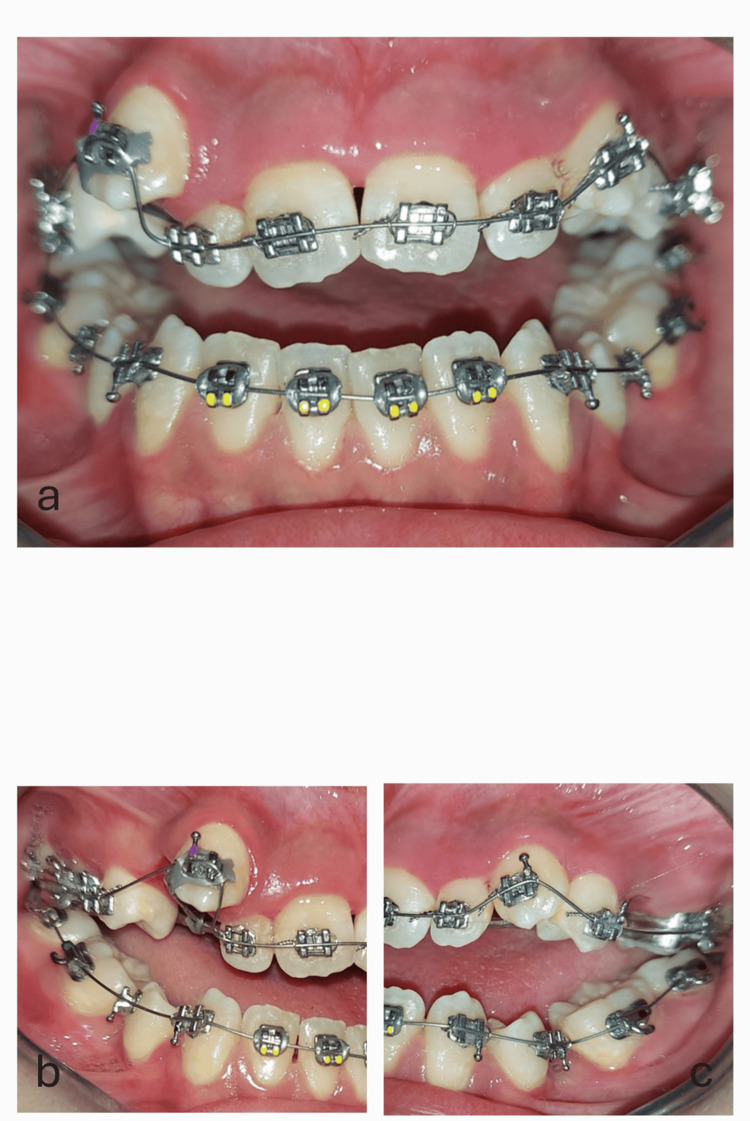
Pre-surgical orthodontic preparation. (a) Frontal occlusal view showing closing ligatures on the maxillary canines and bonding of tooth 12 into the arch, and (b, c) right and left lateral occlusal views showing space management at the 14 and 25 extraction sites and canine retraction mechanics.

The surgical phase consisted of a combined bimaxillary orthognathic procedure. A Le Fort I osteotomy was performed with a 4 mm maxillary advancement and a 2 mm posterior impaction of the maxilla to correct the maxillary retrognathism while controlling the increased lower facial height through a counterclockwise autorotation of the mandible, thereby improving the vertical pattern. Simultaneously, to counteract the sagittal advancement of the chin induced by this autorotation and to definitively correct the sagittal Class III skeletal relationship, a BSSO of the mandible (Obwegeser-Dal Pont technique) was performed for a 3 mm mandibular setback. Rigid internal fixation was successfully achieved using titanium I-plates and osteosynthesis screws for both arches to ensure postoperative stability.

The post-surgical orthodontic phase focused on intercuspation and finishing of the occlusion. First-order wire bends were meticulously incorporated during this phase to optimize tooth positioning, manage finishing mechanics, and ensure the complete closure of all residual micro-spaces. This was followed by debonding and the placement of fixed retainers (T1).

At the end of active treatment, extraoral examination showed a markedly improved facial profile with a harmonious reduction of the lower facial height, enhanced facial proportions, and significantly improved smile esthetics and labio-mental relationship (Figure 6).

**Figure 6:**
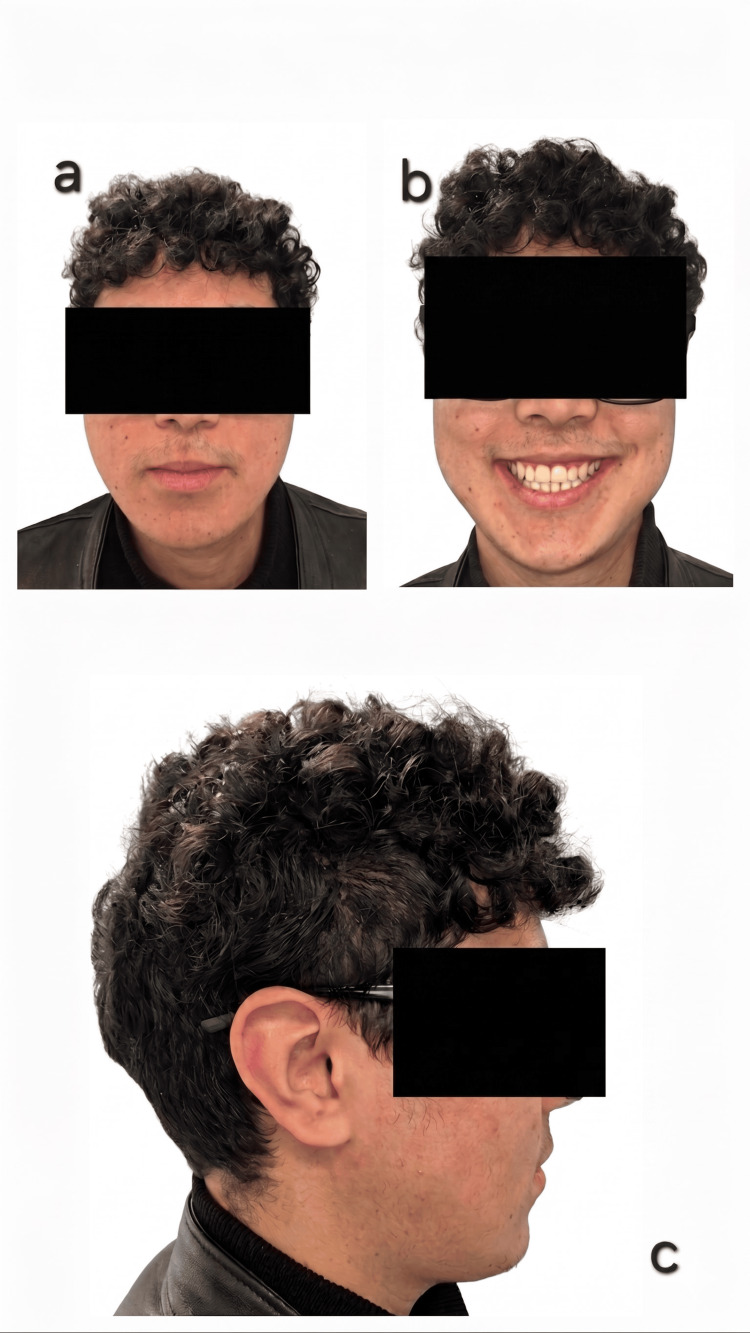
Post-treatment (T1) extraoral examination. (a) Frontal view at rest, (b) frontal view with smile, and (c) right profile view, showing a markedly improved facial profile and enhanced proportions. The patient provided written and signed consent, allowing publication of this identifiable facial image in an open-access journal.

Intraorally, a stable and functional Class I canine and molar relationship was achieved bilaterally (Figures 7a, 7b), with complete correction of the anterior crossbite and normalization of both overjet and overbite (Figure 7c). The maxillary and mandibular arches demonstrated excellent alignment, ideal intercuspation, and optimal coordination, with complete closure of the previous extraction sites (Figures 7d, 7e).

**Figure 7:**
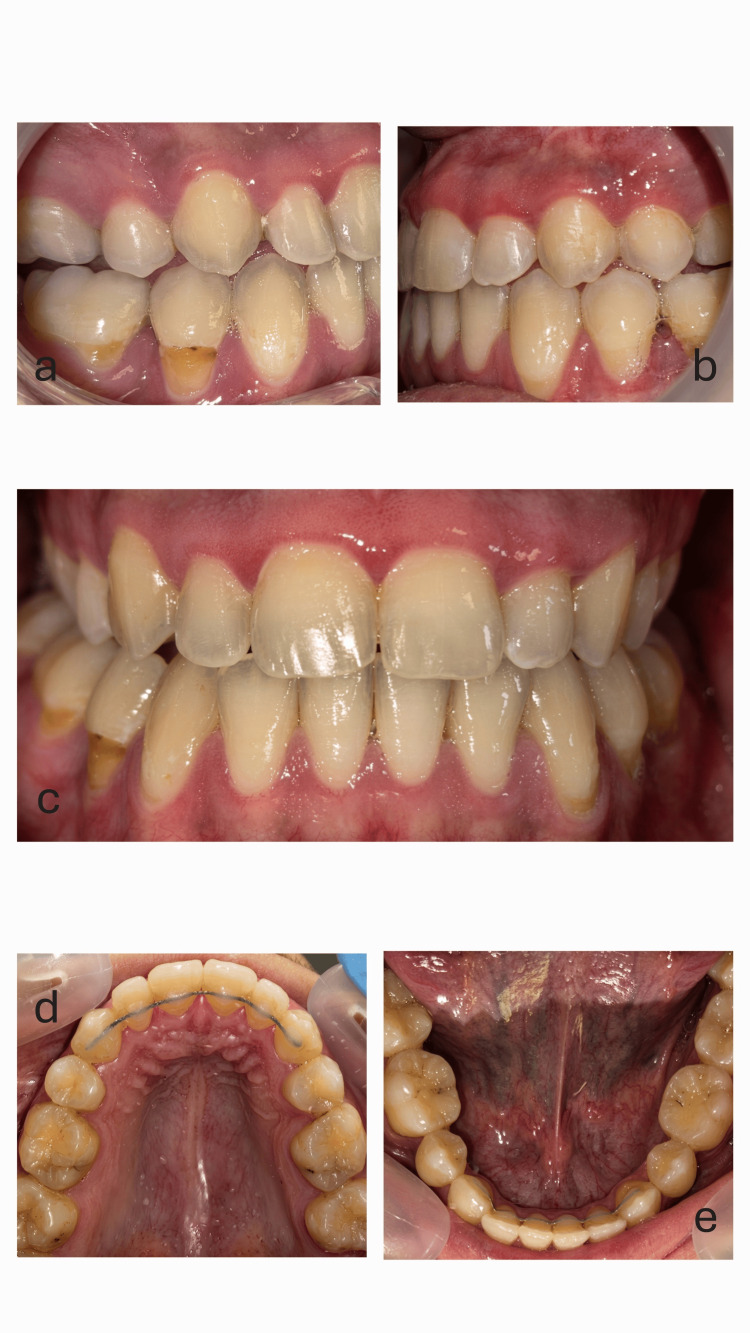
Post-treatment (T1) intraoral views. (a, b) Right and left lateral occlusal views showing a stable Class I canine and molar relationship, (c) frontal occlusal view showing corrected anterior crossbite, (d) maxillary occlusal view, and (e) mandibular occlusal view showing complete space closure and bonded lingual retainer.

The post-treatment panoramic radiograph and lateral cephalogram confirmed satisfactory root parallelism, complete bone fill at the extraction and osteotomy sites, and an intact periodontal status (Figures 8a, 8b). These radiographic exposures were taken just before debonding, with the multibracket appliance still in place.

**Figure 8:**
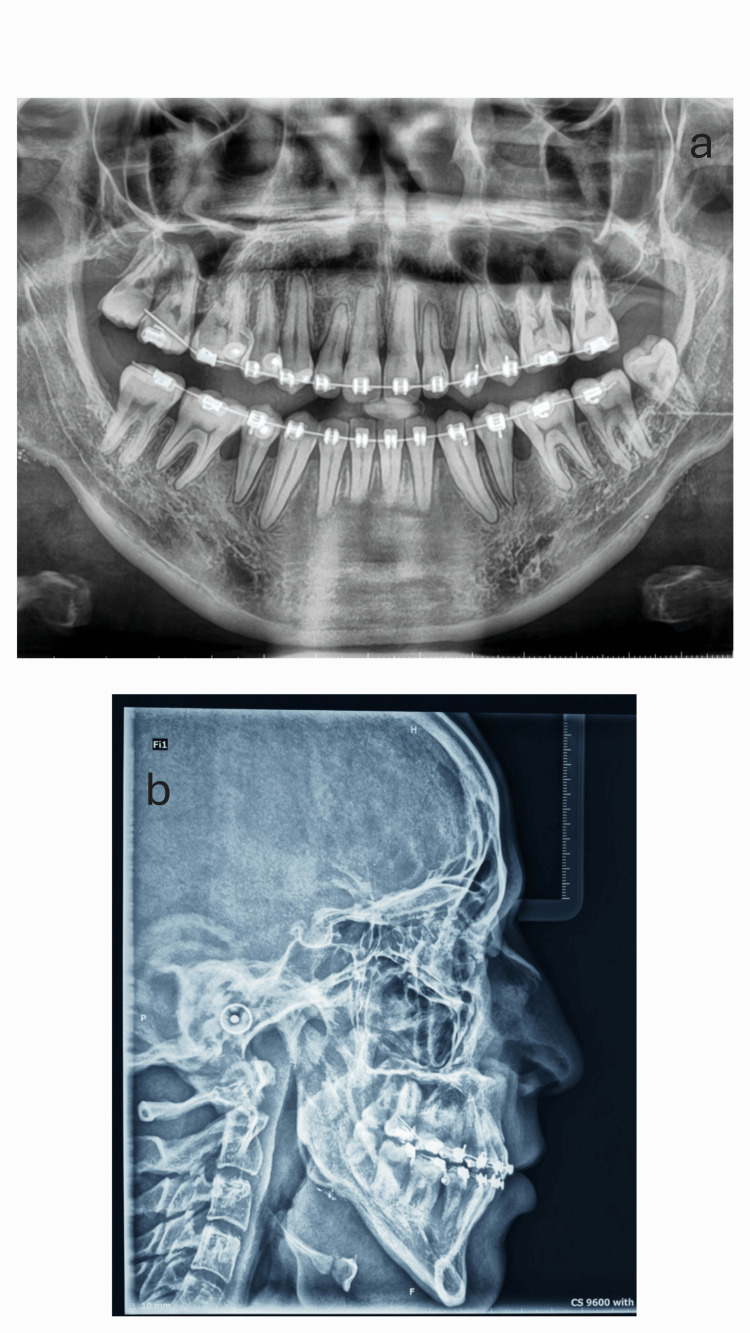
Post-treatment (T1) radiographic examination, taken just before debonding. (a) Panoramic radiograph showing root parallelism and bone fill at the extraction and osteotomy sites, and (b) lateral cephalogram.

Post-treatment cephalometric analysis (Table 3) confirmed a successful correction of the sagittal skeletal discrepancy, with the ANB angle improving from -3° to 3.9° and the AoBo value from -8 mm to -2 mm. A significant reduction in vertical hyperdivergence was also achieved, with the FMA decreasing from 47° to 42° and the GoGn-SN angle from 41° to 40°. Maxillary incisor proclination was successfully controlled (1 to NA reduced from 8 mm/27° to 6 mm/25.5°), while the mandibular incisor inclination was maintained within an acceptable functional range (IMPA adjusted from 75° to 88°). Finally, the interincisal angle (1 to 1) returned to normal limits (131°), reflecting a well-coordinated and stable dento-skeletal correction.

**Table 3:** Comparative cephalometric analysis at T0 (pre-treatment) and T1 (post-treatment, end of active treatment/retention).

Cephalometric variable	Norm (mean ± SD)	T0 (pre-treatment)	T1 (post-treatment)
SNA (°)	82 ± 2	79°	83.2°
SNB (°)	80 ± 2	82°	79.3°
ANB (°)	2 ± 2	-3°	3.9°
AoBo (mm)	0 ± 2	-8 mm	-2 mm
1 to NA (°)	22°	27°	25.5°
1 to NA (mm)	4 mm	8 mm	6 mm
1 to NB (°)	25°	22°	22.7°
1 to NB (mm)	4 mm	6 mm	4.5 mm
1 to 1 (°)	131°	134°	131°
Pog to NB (mm)	0 mm	0 mm	0 mm
GoGn-SN (°)	32 ± 5	41°	40°
FMA (°)	25 ± 3	47°	42°
FMIA (°)	67 ± 3	58°	50°
IMPA (°)	88 ± 3	75°	88°
Angle Z (°)	73°	66°	71°

## Discussion

This case illustrates the diagnostic and therapeutic management of a severe hyperdivergent skeletal Class III malocclusion combining maxillary retrognathism, maxillary endognathia, and significant dento-alveolar compensation (maxillary proalveolia and mandibular retroalveolia). The multi-dimensional severity of the skeletal discrepancy and the high vertical growth pattern clearly justified the indication for a combined surgical-orthodontic treatment rather than orthodontic camouflage [4,7].

The combination of a Le Fort I osteotomy with posterior impaction and a bilateral sagittal split osteotomy (BSSO) for mandibular setback is a well-established approach in hyperdivergent Class III patients [5,6]. Posterior maxillary impaction promotes mandibular counterclockwise autorotation, which significantly improves the vertical pattern and reduces the increased lower facial third height. Although this autorotation would biomechanically worsen the sagittal Class III discrepancy by projecting the chin forward, the concomitant surgical mandibular setback effectively neutralizes this effect to establish an optimal sagittal relationship. In the present case, the severe vertical hyperdivergence was reflected by the pre-treatment cephalometric parameters (FMA = 47°, GoGn-SN = 41°). This high mandibular plane angle pattern represents a severe vertical skeletal discrepancy that could not have been adequately corrected by a mandibular setback alone, fully justifying the vertical control achieved via the bimaxillary procedure.

Pre-surgical decompensation of the maxillary and mandibular incisors is a crucial step in surgical-orthodontic treatment, as it determines the magnitude of achievable surgical movements as well as the final aesthetic and functional outcome [3]. In this patient, the maxillary proalveolia (1 to NA = 8 mm) and the retroinclination of the mandibular incisors (IMPA = 75°) had to be corrected during the pre-surgical phase to allow for appropriate surgical correction without compromising the dento-alveolar articulation.

The prior extraction of the mandibular second premolars required additional consideration in the space management plan. Rather than being treated in isolation, this preexisting space loss was integrated into the calculation of the arch discrepancy (23 mm), which also took into account the incisor decompensation needs and the planned Class III correction. This integration allowed for the efficient use of residual extraction spaces for decompensation and arch coordination, thereby avoiding additional extractions.

Beyond their integration into the space analysis, these imposed extractions raised several specific challenges that warrant discussion. First, the extraction sites for teeth 35 and 45 were not planned according to the surgical objectives of the case. In a growing patient presenting with a hyperdivergent Class III malocclusion requiring a mandibular setback, the choice and timing of mandibular extractions directly influence the degree of incisor decompensation that can be achieved and, consequently, the magnitude of the BSSO movement that can be performed without compromising the periodontal support of the incisors or inducing undesirable changes in the mandibular arch form [3,7]. Since the extraction sites were already defined during the initial consultation, the treatment plan had to be developed a posteriori based on fixed anatomical constraints rather than designed prospectively, which reduced the flexibility usually available when extractions are selected within a scheduled orthodontic-surgical sequence.

Second, at the time of consultation, partial spontaneous space closure and mesial migration of the adjacent posterior segments next to sites 35 and 45 had already occurred. This complicated the calculation of the true residual arch-length discrepancy and required a precise distinction between the space actually available for decompensation and the space already occupied by uncontrolled tooth movements. The mandibular space analysis was thus considerably more complex compared to a case where extractions are performed under direct orthodontic control, since the repositioning index had to be calculated on an arch already in a state of unguided partial adaptation [4].

Third, the absence of the second premolars altered the biomechanical anchorage available in the posterior mandibular segments during the pre-surgical decompensation phase. With one less tooth per quadrant to distribute anchorage strain, increased attention was required to control undesirable mesial movements of the first molars and avoid additional loss of arch length during incisor decompensation, particularly given the severity of the mandibular incisor retroclination (IMPA = 75°) that needed to be corrected prior to surgery [3]. Finally, from a surgical standpoint, the presence of preexisting edentulous spaces close to the planned osteotomy lines for the BSSO required careful attention during pre-surgical planning to ensure that final tooth positions and radicular angulations adjacent to these spaces did not interfere with the osteotomy design or compromise bone-to-bone contact at the osteotomy sites [6].

These considerations demonstrate that extractions performed prior to referral for combined surgical-orthodontic treatment, even when they seem favorable in terms of available space, can constrain treatment planning and increase the complexity of pre-surgical decompensation in severe hyperdivergent Class III cases. This highlights the importance of an early multidisciplinary evaluation, ideally before any extraction, in patients with a suspected skeletal indication for orthognathic surgery [3].

Maxillary endognathia (transverse maxillary deficiency) was managed with a pre-surgical maxillary expander, a step frequently necessary in skeletal Class III patients to prevent post-surgical transverse relapse and to allow for proper coordination of the maxillary and mandibular arches after sagittal correction [2].

The clinical examination at T1 demonstrated a stable Class I occlusion, normalization of the overjet and overbite, and a marked improvement in the facial profile, consistent with previously reported outcomes for combined surgical-orthodontic treatment of severe hyperdivergent skeletal Class III malocclusions [3,5,6]. Long-term follow-up will be required to confirm the stability of the skeletal correction and the dento-alveolar compensation achieved.

## Conclusions

This case highlights the value of a comprehensive cephalometric, dento-alveolar, and difficulty-index-based assessment in planning the surgical-orthodontic management of severe hyperdivergent skeletal Class III malocclusion associated with maxillary retrognathism, maxillary endognathia, and maxillary proalveolia. Combined Le Fort I osteotomy with posterior impaction and BSSO for mandibular setback, supported by adequate pre-surgical decompensation and integration of pre-existing extraction spaces into the space analysis, allowed the achievement of a stable Class I occlusion and a markedly improved facial profile.
